# Identification and validation of a 4-extracellular matrix gene signature associated with prognosis and immune infiltration in lung adenocarcinoma

**DOI:** 10.1016/j.heliyon.2024.e24162

**Published:** 2024-01-09

**Authors:** Yanfei Chai, Yuchao Ma, Wei Feng, Hong Xiang, Hongwei Lu, Longyu Jin

**Affiliations:** a Department of Health Management Center, The Third Xiangya Hospital of Central South University, Changsha, China; b Department of Cardiothoracic Surgery, The Third Xiangya Hospital of Central South University, Changsha, China; c Center for Experimental Medicine, The Third Xiangya Hospital of Central South University, Changsha, China

**Keywords:** Extracellular matrix, Lung adenocarcinoma, Prognosis, Immune microenvironment, Somatic mutation, Therapy response

## Abstract

**Background:**

The extracellular matrix (ECM) plays a crucial role in the development and tumor microenvironment of lung adenocarcinoma (LUAD). This study aimed to establish a risk score of ECM-related genes in LUAD and explore the association between the risk score and patient survival as well as immune cell infiltration, somatic mutations, and therapy response.

**Methods:**

Gene expression data from The Cancer Genome Atlas (TGCA) and eight Gene Expression Omnibus (GEO) databases were used to analyze and identify differentially expressed genes (DEGs). Prognostic ECM-related genes were identified and utilized to formulate a prognostic signature. A nomogram was constructed using TCGA dataset and validated in two GEO datasets. Differences between high- and low-risk patients were analyzed for function enrichment, immune cell infiltration, somatic mutations, and therapy response. Finally, Quantitative real-time PCR (qRT-PCR) was used to detect the mRNA expression of DEGs in LUAD.

**Results:**

A risk score based on four ECM-related genes, *ANOS1*, *CD36*, *COL11A1*, and *HMMR*, was identified as an independent prognostic factor for overall survival (OS) compared to other clinical variables. Subsequently, a nomogram incorporating the risk score and TNM staging was developed using the TCGA dataset. Internal and external validation of the nomogram, conducted through calibration plots, C-index, time-dependent receiver operating characteristics (ROC), integrated discrimination improvement (IDI), and decision curve analyses (DCA), demonstrated the excellent discriminatory ability and clinical practicability of this nomogram. The risk score correlated with the distribution of function enrichment, immune cell infiltration, and immune checkpoint expression. More somatic mutations occurred in the high-risk group. The risk score also demonstrated a favorable ability to predict immunotherapy response and drug sensitivity.

**Conclusion:**

A novel signature based on four ECM-related genes is developed to help predict LUAD prognosis. This signature correlates with tumor immune microenvironment and can predict the response to different therapies in LUAD patients.

## 1 Introduction

Lung cancer is the most common cancer worldwide with a high mortality [1,2]. Lung adenocarcinoma (LUAD) accounts for 40 % of non-small cell lung cancer (NSCLC) and has a 5-year overall survival (OS) of 29.8 % [3]. At present, the American Joint Committee on Cancer tumor-node-metastasis (TNM) staging system is the main approach for evaluating the prognosis of lung cancer. However, LUAD exhibits high heterogeneity in biomarkers of gene mutations and molecular phenotypes. Therefore, it is important to explore new prognostic biomarkers to predict LUAD outcome.

The extracellular matrix (ECM) is an intricate network of secreted macromolecules such as collagens, enzymes, and glycoproteins, whose main function is to provide structural scaffolding and biochemical support for cells and tissues. As an important part of the tumor microenvironment (TME), ECM biosynthesis is regulated by tumor cells, stromal cells (e.g., fibroblasts), and infiltrating immune cells (e.g., macrophages) through mutated genes, transcription factors, signaling pathways, and receptors [4]. These TME-associated cells promote ECM remodeling through increased ECM deposition, fiber alignment, and crosslinking, thereby modifying TME stiffness [5]. Moreover, the components and properties of the ECM can affect the cellular behavior of immune cells and tumor cells by activating or suppressing intracellular signaling processes [4].

Previous research has found that signature derived from bioinformatics analysis has better prognostic performance than single gene. Some studies have also found more effective prognostic models based on different gene sets in LUAD, such as immune checkpoints [6], inflammatory response [7], TME [8] and ECM [9]. However, some studies used only two datasets in the analysis, so their prediction performance may be limited and cannot be well applied to other data. In addition, external validation is also important to improve the universal applicability of these developed signatures. Many studies have attempted to understand ECM-related genes and their roles and mechanisms in the development and progression of LUAD, but there is still a long way to go in this regard. In this study, we aimed to identify potential ECM-related prognostic genes through an analysis of the Cancer Genome Atlas (TCGA) and Gene Expression Omnibus (GEO) databases. We constructed a risk score and prognostic model to assess the prognosis of LUAD and further investigated its association with the tumor immune microenvironment.

## 2 Materials and methods

### 2.1 Data source and analysis

**Fig. 1:**
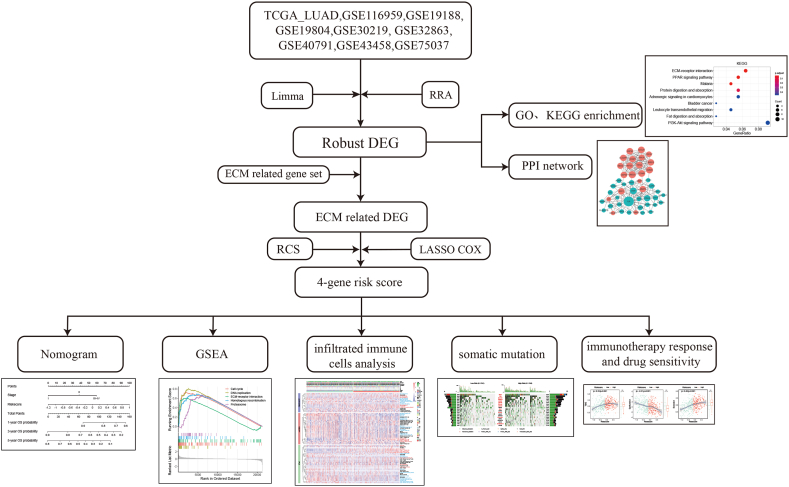
Flow chart of the study.

The overall study design is depicted in Fig. 1. Transcriptomic profiles (raw read counts format) and clinical data of LUAD patients were downloaded from the Genomic Data Commons Data Portal (https://portal.gdc.cancer.gov/) through the “TCGAbiolink” package in R software. 533 tumor tissues and 59 normal tissues were obtained from the TCGA dataset. The data (counts format) were transformed to transcripts per million (TPM) format and normalized by log2(TPM+1). Patients with OS < 0 days or incomplete TNM staging were excluded for survival analysis. Missing data for age, sex, tumor location, and smoking history were complemented by multiple imputation through the “VIM” package [10].

Gene expression data of GSE116959, GSE19188, GSE19804, GSE30219, GSE32863, GSE40791, GSE43458, GSE75037, GSE72094, and GSE68465 were acquired from the GEO database (https://www.ncbi.nlm.nih.gov/geo/) through the “GEOquery” package.

### 2.2 Identification of differentially expressed genes (DEGs)

DEG analysis was performed between tumor tissues and normal tissues using the “limma” package. Genes with |Log2 fold change (FC)| > 1.5 and a Benjamini–Hochberg adjusted p-value <0.01 were defined as DEGs. These DEGs in each dataset were ranked by FC values of up- or down-regulated genes, respectively. Robust DEGs with adjusted p-values <0.01 were then identified using the robust rank aggregation (RRA) algorithm with the “RobustRankAggreg” package. Heatmap was used to describe the top 20 upregulated and 20 downregulated DEGs in each dataset [11].

### 2.3 Gene ontology (GO) functional and Kyoto encyclopedia of genes and genomes (KEGG) pathway enrichment analyses

To determine the biological annotations of robust DEGs, GO functional and KEGG pathway enrichment analyses were conducted and visualized through the “clusterProfiler” package [12]. An adjusted p-value <0.05 was considered statistically significant difference.

### 2.4 Protein–protein interaction (PPI) network of robust DEGs

The “STRINGdb” package was used to predict the PPI of DEGs in the STRING database (http://string-db.org/) [13] with a confidence cutoff of 0.4. The PPI network was visualized with the Cytoscape software (Version 3.90, http://www.cytoscape.org/). The CytoHubba plugin in Cytoscape provides 12 shortest path-based topological analysis methods, including Degree, Edge Percolated Component, Maximum Neighborhood Component, Density of Maximum Neighborhood Component, Maximal Clique Centrality, Clustering Coefficient, and six centralities (Bottleneck, EcCentricity, Closeness, Radiality, Betweenness, and Stress) [14]. The scores of these algorithms were also calculated and ranked by the RRA algorithm. DEGs with adjusted p-values <0.05 were identified as hub genes.

### 2.5 Identification and validation of an ECM-related prognostic signature

696 ECM-related genes were downloaded from the GSEA website (http://www.gsea-msigdb.org/) [15] with the keyword “extracellular matrix”.

Gene expression in each sample was transformed into a Z-score by applying the R function “scale(.)”. This transformation obtained a standard normal distribution (mean = 0, standard deviation = 1) for each gene in different datasets. The endpoint was OS, and progression-free survival (PFS) was also explored if available. Overall survival was defined as time from diagnosis to any cause of death and the censored time is from date of initial diagnosis until the date of last contact. PFS was defined as time from diagnosis to the first occurrence of a new tumor and patients who were alive without the event, or died without tumor were censored [16]. Restricted cubic splines (RCS) were used to explore the linear effect of gene expression on OS. When gene expression had a purely linear effect on OS, it was considered a continuous variable in further analysis. Otherwise, the R function “surv_cutpoint(.)” was used to determine the optimal cutpoint for each variable on OS, and gene expression was divided to two groups (high and low) as binary variables.

Least Absolute Shrinkage and Selection Operator (LASSO) Cox regression was conducted on ECM-related DEGs using the “glmnet” package to minimize the risk of overfitting [17]. The optimal penalty parameter lambda was confirmed by 10-fold cross-validation. Potential prognostic genes were screened based on the optimal lambda and then entered into a univariate Cox regression model through the “survival” package [18]. Prognostic genes with p-values <0.05 were entered into a backward stepwise Cox regression, selecting variables based on the smallest Akaike information criterion (AIC) value through the use of the step function in the "stats" package. The risk score was calculated by the formula:

$$\text{Risk}\;\text{score}=\sum_{i=1}^{n}\beta i\times Expi$$

*βi* represents the coefficient of each gene in the final Cox model, and Expi denotes the gene expression value [19,20]. The gene expression is either a normalized (mean = 0, SD = 1) or dichotomized (0 or 1) value.

Patients were stratified into high- and low-risk groups based on the median risk score (−0.15276). Kaplan-Meier survival analysis with the log-rank test was performed to compare OS between the two groups. Principal component analysis (PCA) and t-distributed stochastic neighbor embedding (t-SNE) were conducted to examine the distribution of the two groups using the "princomp" and "Rtsne" packages, respectively. Moreover, the survival effect of risk scores in LUAD patients stratified by different clinical variables was evaluated using forest plots.

Two GEO datasets, GSE72094 and GSE68465, were used for external validation to explore the independent prognostic value of the signature.

The prognostic ability of the risk score was compared with three published models, including Sun et al. [6], Zou et al. [7] and Xiao et al. [9], using “timeROC” package [21] and Harrell's concordance index (C-index).

### 2.6 Construction and validation of a nomogram

Univariate and multivariate Cox regression analyses were employed to identify independent prognostic factors (i.e., age, sex, smoking history, lateral, T status, N status, M status, clinical stage, and risk score) in TCGA dataset. For continuous variables age and risk score, linear effects were analyzed using RCS as previously mentioned. Based on the Cox regression model, prognostic nomograms of 1-, 3-, and 5-year OS probability were constructed using the “rms” package. The “nomogramEx” package was used to extract polynomial equations to calculate the points of each variable.

The discriminatory performance of the built nomogram model was evaluated using C-index and integrated discrimination improvement (IDI). Decision curve analyses (DCA) was calculated with the source file “stdca.R” to assess the advantage of the nomogram over the TNM staging system. The area under the curve (AUC) of the time-dependent receiver operating characteristics (ROC) for each month was calculated by “timeROC” package and compared with the TNM staging system.

Internal validation for the nomogram was conducted with 1000 bootstrap resamples. A calibration plot was generated to assess the concordance between predicted and actual OS probabilities. GSE72094 and GSE68465 were also used for external validation.

### 2.7 Gene set enrichment analysis (GSEA)

DEGs between high- and low-risk groups were identified using the “limma” package and ranked by log2FC. GSEA was performed using the “gsKEGG” and “GSEA” formulas in the “clusterprofiler” package. The Hallmark (v7.5.1) gene sets were downloaded from the GSEA website (http://www.gsea-msigdb.org/). Gene sets with normalized enrichment score >2 and false discovery rate (q) < 0.05 were considered as statistically significant.

### 2.8 Immune score analysis

Immunedeconv package were applied to quantify the levels of immune cell infiltration for each sample [22]. In this package, 7 algorithms were used to estimating immune cell contents of tumor samples, including CIBERSORT [23], xCell [24], TIMER [25], MCP-counter [26], EPIC [27], quanTIseq [28] and ESTIMATE algortihm [29]. ssGSEA was conducted using the “GSVA” package [30]. The relative gene sets of 28 immune cell subsets were obtained from previous studies [31]. The distribution of immune scores in high- and low-risk groups was compared by the Wilcoxon test and visualized by boxplot. The Shapiro-Wilk test of normality was performed for each variable. Correlation coefficients ρ and p-values between prognostic gene expression or risk scores and immune scores were estimated using the Spearman method if the variables did not follow an independent normal distribution, otherwise Pearson correlation was used.

### 2.9 Tumor mutation status analysis

Somatic mutation information in TCGA patient samples was downloaded from the Genomic Data Commons Data Portal. The maftools package [32] was used to analyze gene mutations between high- and low-risk groups. Mutated genes observed in at least 5 samples in each group were included in the analysis. Tumor mutational burden (TMB) was estimated using tumor-specific somatic mutations.

### 2.10 Prediction of therapeutic sensitivity

Tumor immune dysfunction and exclusion (TIDE) score [33] and immunophenoscore (IPS) were employed to assess the potential response to immunotherapy for each patient. The TIDE scores were calculated by the TIDE website (http://tide.dfci.harvard.edu). IPS data were download from The Cancer Immunome Atlas database (https://tcia.at/home). The differences of TIDE score and IPS between high- and low-risk groups were analyzed by Wilcox test. Generally, a lower TIDE score and a higher IPS predict a better response to immunotherapy. The differential expression of immune checkpoints between high- and low-risk groups was also investigated.

The “oncoPredict” R package was used to predict clinical response to chemotherapy. The Genomics of Drug Sensitivity in Cancer 2 data [34] prepackaged into the “oncoPredict” was used as the training dataset, which contains large-scale gene expression of 805 samples and drug screening data of 198 drugs. The gene expression matrix in TGCA-LUAD was used as the testing dataset and the half maximal inhibitory concentration (IC50) value of the 198 drugs in each patient was calculated.

### 2.11 Tissue samples

15 primary tumor tissues and corresponding adjacent normal tissues were obtained from patients with LUAD at the Third Xiangya Hospital of Central South University. The clinical characteristics of the 15 patients were shown in Table S1. None of the patients had received chemotherapy, radiotherapy, target therapy or immunotherapy. The study received approval from the Human Research Ethics Committee of the Third Xiangya Hospital of Central South University.

### 2.12 Quantitative real-time PCR (qRT-PCR)

Total RNA from tissues was extracted using Trizol reagent (Invitrogen, USA) following the manufacturer's instructions. The process of cDNA synthesis utilized the Evo M-MLV RT Mix Kit (Accurate Biology, China), followed by qRT-PCR analysis using the SYBR Green premix qPCR Kit (Accurate Biology, China). The procedure was executed on the Roche LightCycler 480 II instrument (Roche, Basel, Switzerland). ACTB was used as internal controls for the normalization. Subsequently, the relative mRNA expression levels were determined utilizing the 2-ΔΔCt method. The primer sequences are shown in Table S2.

### 2.13 Statistical analyses

All statistical analyses were conducted using R (version 4.2.2) and its packages, such as ggplot2, ggpubr, ggExtra, ggrepel, survival, survminer, finalfit, and dplyr. A two-tailed p-value <0.05 was regarded as statistically significant if not specifically mentioned. When indicated, p-values were adjusted using the Benjamini–Hochberg methods. The identification of DEGs was carried out utilizing the "limma" package. Independent prognostic factors were discerned through a combination of LASSO Cox analyses, as well as univariate and multivariate Cox analyses. The Kaplan-Meier plots for survival probabilities were employe for prognostic prediction, and the log rank test was applied to test the difference in survival prognosis [35,36]. The Wilcoxon test was utilized to assess and compare immune cell infiltration, TMB, expression of immune checkpoints, and drug sensitivity levels between the low- and high-risk groups. Correlations coefficients were assessed using Spearman or Pearson correlation analysis.

## 3 Results

### 3.1 Identification of DEGs

**Fig. 2:**
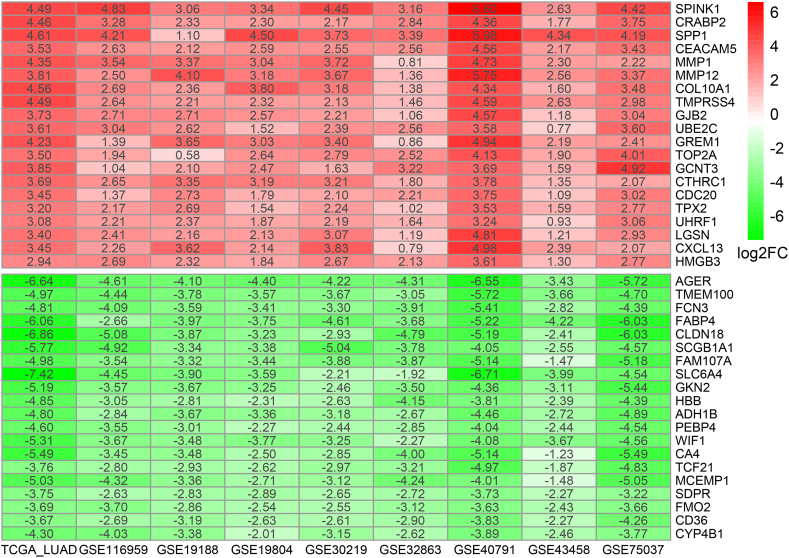
Heatmap of the top 20 upregulated and downregulated DEGs identified by the RRA algorithm in LUAD across nine datasets. Red indicates upregulated DEGs, while green indicates downregulated DEGs. Numbers in the cells indicate the log2FC of the gene expression in tumor tissue compare to normal tissue.

In this study, a comprehensive analysis of DEGs in LUAD was carried out using data fromTCGA and eight GEO datasets. The basic information of the nine datasets and related DEGs are present in Table S3. Volcano plots (Fig. S1) described the distribution of DEGs in each dataset. Following the RRA algorithm, a total of 223 robust DEGs were identified, comprising 63 upregulated genes and 160 downregulated genes (Table S4). The heatmap illustrated the log2FC of the leading 20 upregulated and 20 downregulated DEGs in each dataset (Fig. 2).

### 3.2 GO and KEGG enrichment analyses

**Fig. 3:**
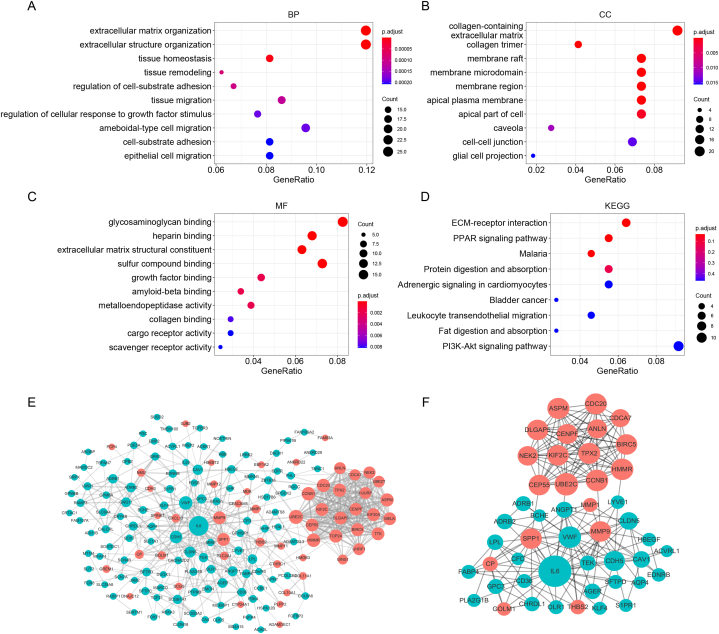
GO and KEGG enrichment and PPI network construction with DEGs. **(A)** Biological process. **(B)** Cellular component. **(C)** Molecular function. **(D)** KEGG pathway enrichment. **(E)** PPI network construction of DEG. **(F)** Hub genes identified by the RRA algorithm.

The GO enrichment analysis, specifically in the biological process category, revealed that the identified robust DEGs were significantly enriched in processes such as “extracellular matrix organization”, “extracellular structure organization”, “tissue homeostasis”, “tissue remodeling”, and “regulation of cell-substrate adhesion”. In the cellular component category, these DEGs showed enrichment in locations such as “collagen-containing extracellular matrix”, “membrane raft membrane microdomain”, “collagen trimer”, and “membrane region”. Moving to the molecular function category, the DEGs demonstrated enrichment in functions including “glycosaminoglycan binding”, “heparin binding”, “extracellular matrix structural constituent”, “sulfur compound binding”, and “growth factor binding”. Furthermore, KEGG pathway analysis highlighted the involvement of the robust DEGs in several pathways. Notable pathways included “ECM-receptor interaction”, “PPAR signaling pathway” and “Malaria” (Fig. 3A–D, Table S5).

### 3.3 Construction of PPI network

The PPI network included 169 nodes and 304 edges. After calculating the score of each node through 12 topological algorithms using the cytoHubba plugin, 47 hub genes were identified with the RRA algorithm and a PPI network with 199 edges was constructed (Fig. 3E–F).

### 3.4 Identification and validation of an ECM-related prognostic signature

**Fig. 4:**
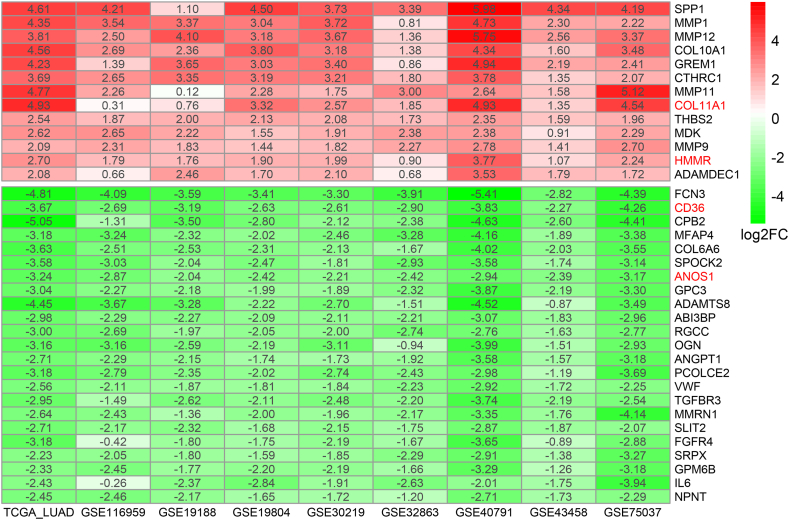
Heatmap of the 13 upregulated and 23 downregulated ECM related genes in LUAD across nine datasets. The gene symbols highlighted in red represent the four genes selected through multivariate Cox analysis.

GO and KEGG analyses demonstrated that robust DEGs were strongly related to ECM. Thus, In the following investigation, we delved into the impact of ECM-related DEGs on patient prognosis and the tumor immune microenvironment. Within the ECM-related gene set, a total of 36 genes were recognized as DEGs, comprising 13 upregulated genes and 23 downregulated genes (Table S6). The heatmap succinctly illustrated the differential expression patterns of these genes across both TCGA and eight GEO datasets, providing a comprehensive overview of their expression profiles (Fig. 4). RCS analysis found that only *HMMR*, *GPC3*, and *MMP1* had a purely linear effect on OS (Table S7, Fig. S2). Thus, these three genes were regarded as continuous variable in subsequent analyses, while the other 33 genes were transformed into binary variables according to the optimal cutoffs.

**Fig. 5:**
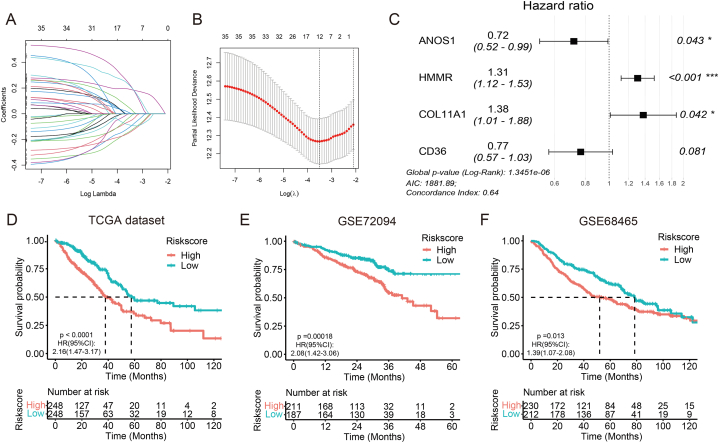
Construction and validation of ECM-related signature. **(A, B)** Lasso Cox analysis identified ECM-related genes associated with prognosis in the TCGA dataset. **(C)** Forest plot by multivariate Cox analysis to evaluate the prognostic values of four ECM genes. **(D**–**F)** Kaplan–Meier plot revealed that OS was higher in patients with low risk scores compared to those with high risk scores in TCGA **(D)**, GSE72094 **(E)**, and GSE68465 **(F)**. *p < 0.05; ***p < 0.001.

ECM-related DEGs underwent further scrutiny through LASSO Cox regression analysis, resulting in the identification of 12 genes with the minimum λ value (Fig. 5A–B). To assess their prognostic significance, univariate Cox regression was employed, leading to the inclusion of 9 genes in a subsequent backward stepwise multivariate Cox regression analysis. Table S8 shows the detailed results of univariate and multivariate Cox regression. Based on the minimum AIC value (AIC = 1881.89) in the model (Fig. 5C), the final risk score was calculated as follows:

riskscore=ANOS1×−0.33050+CD36×−0.26597+HMMR×0.26731+COL11A1×0.31983

The patients were then categorized into high- and low-risk groups based on the median risk score. Table 1 depicts the clinical and pathological characteristics of the two groups of patients in TCGA dataset. Kaplan-Meier curve demonstrated a significantly longer OS for patients with low risk scores (Fig. 5D) and a similar trend for PFS (Fig. S3A). Patients were also divided into two groups according to the risk score as well as PCA and t-NSE (Fig. S4). Subgroup analysis verified the favorable prognostic value of the risk score across various clinical and pathological features (Fig. S5).

**Table 1:** The clinical and pathological characteristics of LUAD patients in the TCGA dataset.

Variable	Low-risk	High-risk	p
	N (%)	N (%)	
ALL	248	248	
Age (mean (SD))	65.8 (9.4)	64.6 (10.5)	0.159
Age			
≤65	134 (54.0)	121 (48.8)	0.281
>65	114 (46.0)	127 (51.2)	
Sex			
Female	145 (58.5)	121 (48.8)	0.038
Male	103 (41.5)	127 (51.2)	
Smoking			
Yes	206 (83.1)	221 (89.1)	0.069
No	42 (16.9)	27 (10.9)	
Location			
LLL	42 (16.9)	37 (14.9)	0.412
LUL	54 (21.8)	64 (25.8)	
RLL	50 (20.2)	43 (17.3)	
RML	8 (3.2)	15 (6.0)	
RUL	94 (37.9)	89 (35.9)	
T status			
T1	98 (39.5)	70 (28.2)	0.069
T2	121 (48.8)	144 (58.1)	
T3	21 (8.5)	24 (9.7)	
T4	8 (3.2)	10 (4.0)	
N status			
N0	184 (74.2)	147 (59.3)	0.003
N1	39 (15.7)	55 (22.2)	
N2	25 (10.1)	44 (17.7)	
N3	0 (0.0)	2 (0.8)	
M status			
M0	241 (97.2)	234 (94.4)	0.181
M1	7 (2.8)	14 (5.6)	
Stage			
I	158 (63.7)	112 (45.2)	<0.001
II	55 (22.2)	69 (27.8)	
III	28 (11.3)	53 (21.4)	
IV	7 (2.8)	14 (5.6)	
Progression			
No	155 (62.5)	136 (54.8)	0.101
Yes	93 (37.5)	112 (45.2)	
Status			
Alive	178 (71.8)	139 (56.0)	<0.001
Dead	70 (28.2)	109 (44.0)	

LLL, left lower lobe; LUL, left upper lobe; RLL, right lower lobe; RML, right middle lobe; RUL, right upper lobe.

The robustness of the risk score's predictive performance was further validated in GSE72094 and GSE68465. Detailed clinical and pathological characteristics of these datasets are presented in Table S9. Consistent with the findings from the TCGA dataset, patients in the high-risk group exhibited significantly better OS compared to those in the low-risk group in both GSE72094 and GSE68465 (Fig. 5E and F). Additionally, in GSE68465, patients with low risk scores also displayed a longer PFS than those with high risk scores (Fig. S3B).

We also assessed the efficacy of the risk score against three established models, including Sun et al. [6], Zou et al. [7] and Xiao et al. [9]. The time-dependent ROC analysis indicated that the predictive ability of this model was not inferior to the previous models (Fig. S6A). Meanwhile, there was no significant difference in Harrell's C-index between this and the other three models (Fig. S6B).

### 3.5 Construction and validation of nomogram

**Fig. 6:**
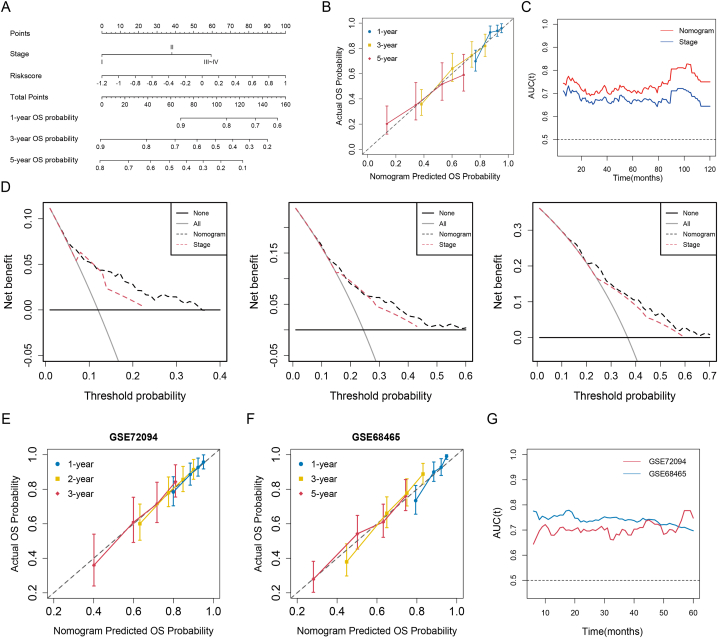
Construction and validation of a nomogram with four ECM-related risk scores. **(A)** A nomogram constructed with risk score and TNM stage predicts the probability of 1, 3, and 5 years of OS probabilities in TCGA dataset. **(B)** Calibration plot assesses the consistency between the actual OS probability and the predicted OS probability of the nomogram in TCGA. **(C)** Time-dependent AUC curves of the nomogram and TNM staging system in TCGA. **(D)** DCA curves of the nomogram for predicting 1- (left), 3- (middle), 5-year (right) OS. **(E, F)** Calibration plots of the nomogram in GSE72094 **(E)** and GSE68465 **(F)**. **(G)** Time-dependent AUC values of the nomogram in GSE72094 and GSE68465.

RCS curves found that risk score had a linear effect on OS, but age did not (Fig. S7). Univariate Cox regression analysis showed that T status, N status, M status, clinical stage, and risk score were all significantly associated with the prognosis. Multivariate Cox regression analysis confirmed that both risk score and staging were independent prognostic factors with the minimum AIC value (AIC = 1841.62) (Table 2). A nomogram was constructed to predict 1-, 3-, and 5-year OS probability in LUAD patients (Fig. 6A). Table S10 shows the calculation formula for each variable.

**Table 2:** Cox regression analysis of prognostic variables for OS in TCGA dataset.

Variable	Univariate analysis		Multivariate analysis	
	HR (95%CI)	p	HR (95%CI)	p
Age (>65y vs ≤ 65y)	1.142 (0.850–1.535)	0.379		
Sex				
(Male vs Female)	1.059 (0.789–1.421)	0.704		
Smoking (Yes vs No)	0.985 (0.639–1.520)	0.947		
Lateral				
(Right vs Left)	1.002 (0.740–1.357)	0.987		
T status		<0.001		
T1	1			
T2	1.455 (1.019–2.078)	0.039		
T3-4	2.986 (1.891–4.716)	<0.001		
N status		<0.001		
N0	1			
N1	2.438 (1.730–3.435)	<0.001		
N2-3	2.964 (2.025–4.338)	<0.001		
M status (M1 vs M0)	2.072 (1.176–3.651)	0.012		
TNM stage		<0.001		<0.001
Stage I	1		1	
Stage II	2.293 (1.600–3.286)	<0.001	2.043 (1.422–2.936)	<0.001
Stage III ∼ IV	3.443 (2.411–4.915)	<0.001	3.049 (2.128–4.369)	<0.001
Risk score	2.718 (1.920–3.848)	<0.001	2.345 (1.636–3.362)	<0.001

The calibration plot demonstrated excellent concordance between the predictions generated by the nomogram and the actual OS probabilities at 1, 3, and 5 years (Fig. 6B). The Harrell's C-index of the nomogram (0.697, 95 % CI: 0.652–0.742) was significantly higher than that of the TNM staging system (0.658, 95 % CI: 0.617–0.699; p < 0.001). Compared with the TNM staging system, the nomogram had an IDI value of 1.80 % (p < 0.001), 3.72 % (p < 0.001), and 3.77 % (p < 0.001) at 1, 3, and 5 years, respectively. Time-dependent ROC also showed that the nomogram had higher AUCs than the TNM staging system in predicting the prognosis of LUAD patients (Fig. 6C). The DCA curves also showed that the new nomogram had better clinical utility than the TNM staging system due to the wide and practical ranges of threshold probabilities (Fig. 6D).

In external validation using the validation datasets GSE72094 and GSE68465, calibration plots showed considerable agreement between the nomogram-predicted OS probabilities and actual OS probabilities (Fig. 6E–F). Time-dependent ROC also showed favorable AUCs of the nomogram in predicting OS probability (Fig. 6G).

### 3.6 GSEA analysis

**Fig. 7:**
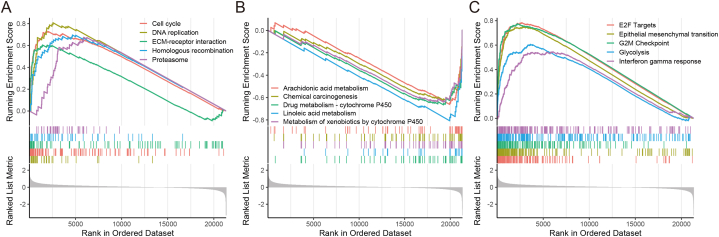
Gene set enrichment analysis in TCGA cohort. Significantly enriched pathways in the KEGG analysis in high-risk group**(A)** and low-risk group**(B)**. Enriched pathways in the Hallmark gene sets between two groups**(C)**.

In the KEGG pathway analysis, gene sets associated with the high-risk group showed enrichment in processes such as cell cycle, DNA replication, ECM-receptor interaction, homologous recombination, and proteasome (Fig. 7A). Conversely, gene sets from the low-risk samples exhibited enrichment in metabolic pathways, including arachidonic acid metabolism, chemical carcinogenesis, drug metabolism-cytochrome P450, linoleic acid metabolism, and metabolism of xenobiotics by cytochrome P450 (Fig. 7B). Regarding hallmark gene sets, enrichment was observed in E2F targets, epithelial-mesenchymal transition, G2M checkpoint, glycolysis, and interferon-gamma response (Fig. 7C).

### 3.7 Association of risk score with tumor immune microenvironment

**Fig. 8:**
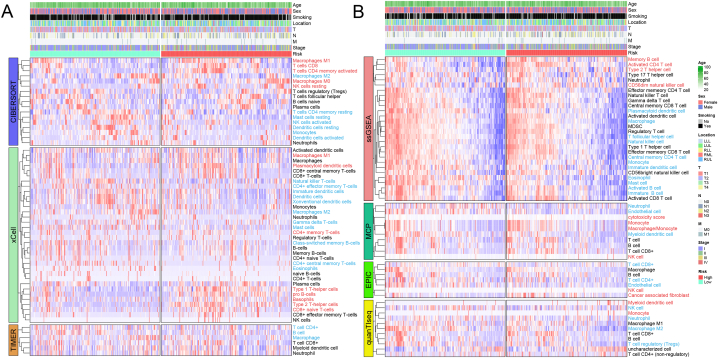
Landscape of immune cell infiltration calculated by (A)CIBERSORT, xCell, TIMER, (B) MCP-counter, EPIC, quanTIseq and ssGSEA in the low- and high-risk groups. Blue and red represent lower and higher immune cell infiltration in the high-risk group, respectively.

The distribution of infiltrating immune cells, estimated by CIBERSORT, xCell, TIMER, MCP-counter, EPIC, quanTIseq, and ssGSEA, in the low-risk and high-risk groups was comprehensively investigated (Fig. 8A–B). The differences in the proportion of each cell type between the low-risk and high-risk groups were illustrated in Fig. S8. The results revealed a significant reduction in various anti-tumor immune cells, including CD4^+^ central memory T cells, dendritic cells, mast cells, natural killer T cells, and monocytes, in the high-risk group, whereas T helper 2 (Th2) cells were increased. Notably, the high-risk group exhibited a higher proportion of M1 macrophages and a lower proportion of M2 macrophages, suggesting a propensity for the M1 phenotype in patients within this group.

Stromal score, immune score, ESTIMATE score, and tumor purity were quantified using the ESTIMATE algorithm. The risk score demonstrated a negative correlation with stromal score, immune score, and ESTIMATE score, while tumor purity was higher in the high-risk group. (Figs. S8A–B).

### 3.8 Mutation status of high- and low-risk patients

**Fig. 9:**
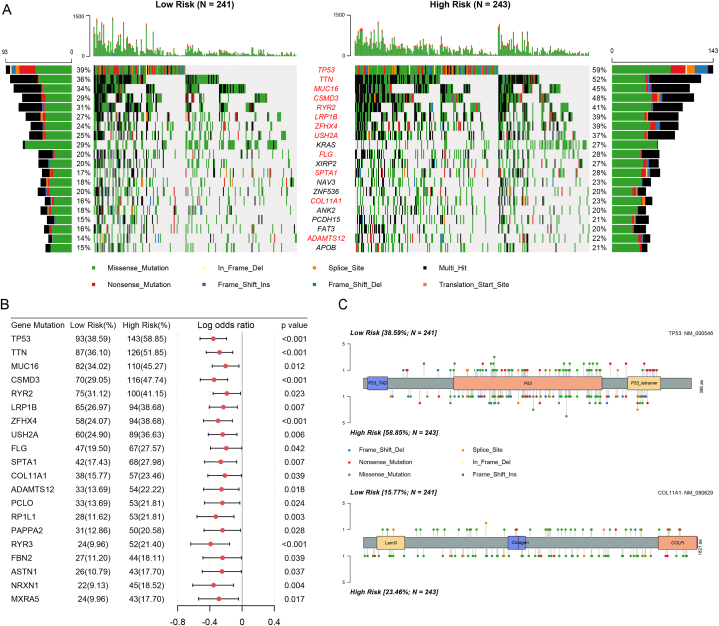
Somatic mutations in the high- and low-risk groups. **(A)** Comparison of the top 20 most frequently mutated genes in the two groups. Red represents a higher frequency of gene mutations in the high-risk group. **(B)** Forest plot of differentially mutated genes in high- and low-risk patients. **(C)** Lollipop chart displaying mutation sites of TP53 and COL11A1 proteins.

Somatic mutations were more prevalent in high-risk patients compared to low-risk patients. Maftools analysis revealed that among the top 20 mutated genes in LUAD patients (Fig. 9A), 12 exhibited higher mutation frequencies in the high-risk group. These genes included *TP53*, *CSMD3*, *TTN*, *ZFHX4*, *USH2A*, *SPTA1*, *LRP1B*, *MUC16*, *ADAMTS12*, *RYR2*, *COL11A1*, and *FLG*. Examination of the top 20 differentially mutated genes between the low- and high-risk groups showed that the mutation frequencies of all these genes were higher in the high-risk group (Fig. 9B). Regarding the most mutated gene, *TP53*, a higher mutation rate was observed in the high-risk group (58.85 %) compared to the low-risk group (38.59 %). Additionally, among the four genes in the 4-gene signature, *COL11A1* was the sole gene with differential mutations, and a higher mutation frequency was observed in the high-risk group (23.46 %) than in the low-risk group (15.77 %) (Fig. 9C).

### 3.9 Prediction of therapeutic sensitivity

**Fig. 10:**
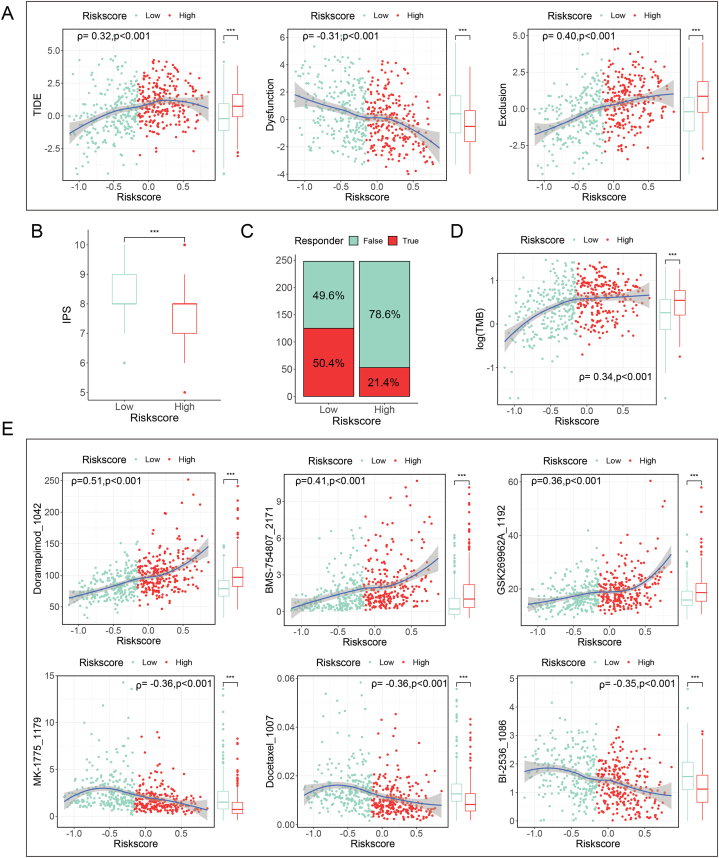
Correlation analysis of risk score and therapeutic sensitivity. **(A)** Correlation of risk score with TIDE score, dysfunction score, and exclusion score. **(B)** Comparison of IPS between high-risk group and low-risk group. **(C)** Distribution of immunotherapy responders in the high- and low-risk groups. **(D)** Correlation between risk score and TMB. **(E)** Correlation between risk score and drug sensitivity.

Regarding the response to immunotherapy, patients in the high-risk group exhibited a higher TIDE score and a lower IPS score (Fig. 10A and B), suggesting poorer responses to immunotherapy in this group (Fig. 10C). TMB, a biomarker of immunotherapy response, was also significantly higher in the high-risk group (Fig. 10D). Additionally, twenty-six immune checkpoints were significantly modulated in the high-risk group (Fig. S9A). The risk score demonstrated a positive correlation with the expression of 10 immune checkpoints, including *PD-L1*, *PD-L2*, *PD-1*, *B7–H3*, and *LAG3* (Fig. S9B), and a negative correlation with the expression of 20 immune checkpoints such as *BTLA*, *CD28*, *CD4*0LG, and *TNFSF15*.

IC50 values for 198 drugs, calculated using “oncopredict” with GDSC as the training data, revealed distinct drug sensitivities in TCGA patients.Specifically, patients in the low-risk group exhibited greater sensitivity to drugs such as doramapimod, IGF1R inhibitor BMS-754807, and ROCK inhibitor GSK269962A, Conversely, patients in the high-risk group demonstrated heightened sensitivity to drugs including WEE1 inhibitor MK-1775, docetaxel, PLK1 inhibitor BI-2536, cisplatin, paclitaxel, and vinorelbine (Fig. 10E–Table S11).

### 3.10 Validation of the four ECM related gene

**Fig. 11:**
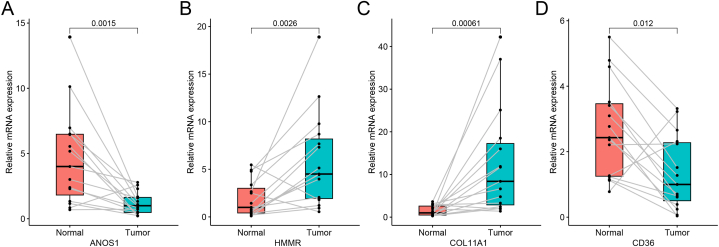
The mRNA expression of four prognostic genes between normal tissues and tumor tissues by qRT-PCR. **(A)***ANOS1*. **(B)***HMMR*. **(C)***COL11A1*. **(D)***CD36*.

The mRNA expression of the four prognostic genes between normal tissues and tumor tissues were verified by qRT-PCR. The result confirmed that *ANOS1* and *CD36* were downregulated in LUAD, while *HMMR* and *COL11A1* were upregulated, which were consistent with the expression differences in nine datasets (Fig. 11A–D).

## 4 Discussion

In this study, we conducted an analysis of ECM-related DEGs in LUAD patients from TCGA and GEO datasets, identified a 4-gene prognostic signature and developed a nomogram to predict patient prognosis. In the 4-gene prognostic signature, *HMMR* and *COL11A1* were upregulated in LUAD, and upregulation of these two genes was associated with worse prognosis, while *CD36* and *ANOS1* were downregulated, and their downregulation was associated with poorer prognosis. This 4-gene prognostic signature was identified as an independent prognostic factor for LUAD. Compared with the previous ECM model, our model used more datasets to screen DEGs and evaluate the prediction performance, and the final prognostic model used fewer genes to achieve similar prediction performance. The nomogram, constructed using the risk score and TNM staging system, demonstrated the ability to predict OS probability in LUAD patients. C-index, time-dependent ROC, IDI, and DCA data all demonstrated that the nomogram has favorable discriminatory ability and clinical practicability compared with the TNM staging system. Further, the nomogram showed considerable calibration accuracy in two externally validated GEO cohorts. Simultaneously, we investigated the association between the risk score and infiltrating immune cell types using multiple algorithms. Our findings indicated that patients in the high-risk group exhibited varying degrees of immune cell infiltration, higher tumor purity, elevated somatic mutation status, and increased TMB compared to those in the low-risk group. Furthermore, the risk score demonstrated a notable capacity to predict both immunotherapy response and chemotherapy sensitivity.

ECM plays a pivotal role in cancer-related processes such as cell proliferation, migration, and angiogenesis [37], In this study, we developed a prognostic model based on four ECM-related genes. Among these genes, *HMMR* acts as a receptor for hyaluronic acid and is closely associated with cell motility. Elevated expression of *HMMR* has been linked to various cancers, including breast [38] and lung cancer [39,40]. In the context of LUAD, *HMMR* is highly expressed and has been shown to promote tumor cell proliferation, migration, and invasion while inhibiting apoptosis [39]. High levels of *HMMR* in primary LUAD are correlated with inflammatory molecular signatures and an unfavorable prognosis. Importantly, LUAD cells lacking *HMMR* expression lose their capacity for tumor initiation and distant metastasis [40].

*CD36* is a heavily glycosylated 88-kD class B scavenger receptor with dual functionality as both a signal transducer and a fatty acid transporter [41]. Despite its role in promoting cell migration, invasion, and metastasis in several cancers such as breast cancer [42], urinary bladder cancer [43], colorectal cancer [44], and gastric cancer [45]. This protein also has an important role in mediating epithelial-to-mesenchymal transition (EMT) through different signaling pathways in various cancers. However, we observed that *CD36* was downregulated in LUAD and was associated with poorer prognosis. Interestingly, our findings align with previous reports indicating that the re-expression of silenced *CD36* inhibits the proliferation, migration, and invasion of lung cancer cells, arresting the cell cycle at the G0/G1 phase [46]. The underlying mechanisms for these effects remain unknown.

*COL11A1* is highly expressed in tumor-associated fibroblasts, which secrete *COL11A1* into the ECM to regulate tumor cell proliferation, angiogenesis, invasion, and drug resistance [[47], [48], [49]]. Previous reports have shown that mutant *COL11A1* promotes tumorigenesis and cancerous invasion in cutaneous squamous cell carcinoma [50]. The same is true for *COL11A1* in LUAD. We observed that *COL11A1* mutations were common in LUAD, especially in the high-risk group.

Knowledge of the role of *ANOS1* in pathophysiology is limited. Mutations in *ANOS1* have been linked to the X-linked Kallmann syndrome. The encoded protein has sequence similarity to proteins that function in neural cell adhesion and axon migration. Moreover, circulating *ANOS1* serves as a diagnostic biomarker for gastric cancer [51], and the protein is involved in brain tumor malignancies through integrin signaling [52]. However, few reports have linked *ANOS1* to lung cancer.

We developed a nomogram incorporating the 4-gene signature and TNM staging, demonstrating robust clinical utility across different LUAD datasets. This suggests that the risk score serves as a valuable complement to the TNM staging system for predicting LUAD prognosis. In GSEA analysis, we observed significant enrichment of tumor-related pathways in the high-risk group, including cell cycle, DNA replication, G2M checkpoint, glycolysis, and interferon (IFN)-γ response. Additionally, ECM-related pathways such as ECM-receptor interaction and EMT were enriched in the high-risk group. Conversely, several metabolism-related pathways were enriched in the low-risk group. These findings indicate that high- and low-risk groups exhibit distinct molecular profiles and specific pathway signatures.

The TME is important in tumorigenesis and tumor immunity. The remodeling of ECM, as an important part of TME, also affects tumor immunity. Stiff extracellular matrix can block the release of cancer cell antigens, reduce cancer antigen presentation, affect the priming, activation, and immigration of effector T cells, and interfere with the recognition and killing of cancer cells by T cells [4]. We confirmed that the risk score was negatively related to immune score and stroma score, which were positively related to prognosis [8]. Our findings indicate a correlation between the risk score and various infiltrating immune cell types. The significant disparities observed in the distribution of several infiltrating immune cell types between the high- and low-risk groups suggest that alterations in ECM composition could impact the tumor immune microenvironment. Th1 cells are generally considered as anti-tumor immune cells that inhibit tumor growth and activate tumor-specific immune mechanisms by producing IFN-γ, TNF-α, and IL-2. Interestingly, we found that Th2 cells were significantly enriched in the high-risk group. Increased Th2 cells promote cancer growth and metastasis by producing IL-4, IL-5, and IL-10 [53]. In human cancers, Th2 cell infiltration in the TME is often associated with poor clinical outcome [54]. We demonstrated that Th2 cells calculated by xCell and ssGSEA were negatively related to LUAD prognosis (Fig. S10 A-B).

Our findings further revealed that individuals in the low-risk group were more inclined to exhibit the M2 phenotype, while most patients in the high-risk group predominantly demonstrated the M1 phenotype. Tumor-associated macrophages typically undergo polarization into two distinct phenotypes: M1 (classically activated) and M2 (alternatively activated). M1 macrophages, activated by IFN-γ, lipopolysaccharides, and TNF-α, play a role in fostering inflammatory responses against tumor cells. Conversely, M2 macrophages, activated by IL-4 and IL-10, contribute to tumor progression and metastasis [55,56]. M1 macrophages are positively associated with the progress of NSCLC [57] and infiltration of M2 macrophages is associated with reduced overall survival [58]. However, some studies also found CD204+ M2 macrophages to be an independent prognosticator of prolonged survival in NSCLC [59]. In this analysis, only M2 score predicted by xCell was positively associated with LUAD prognosis (Fig. S10 C-F), indicating that the predictive value of different algorithms is influenced by variability in the characterization methods of infiltrating immune cell subtypes. In addition, macrophages affect tumor cell invasiveness by remodeling ECM components. Previous studies have showed that macrophage-derived SPARC (secreted protein acidic and rich in cysteine) regulates ECM deposition and cell-ECM interactions and enhances tumor cell invasion and adhesion to other ECM components [60]. Thus, more evidence is still needed to investigate the relationship between macrophage phenotype and ECM remodeling.

Gene mutations play a crucial role in the development of lung cancer. We examined the association between risk scores and genetic mutations, revealing that the most frequently mutated genes in LUAD exhibited higher mutation frequencies in the high-risk group. Mutations in *TP53*, the gene encoding the well-known tumor-suppressor p53 that induces apoptotic cell death in response to oncogenic stress, occur in approximately 50 % of human cancers [61]. We found that 58.85 % of patients in the high-risk group had *TP53* mutations. Mutant p53 displays the ability to promote proliferation, migration, invasion, metastasis, angiogenesis, and tissue remodeling. Whether mutant p53 can alter the composition of ECM remains to be further determined.

Immunotherapy represents a promising avenue for LUAD treatment, with immune checkpoint inhibitors being recommended as first-line therapy for eligible patients with metastatic LUAD. Our analysis revealed a connection between the risk score and the TIDE score and IPS score, indicating that high-risk patients exhibited a diminished response to immunotherapy. Furthermore, we observed significant alterations in the expression levels of numerous immune checkpoints in the high-risk group, suggesting a potential involvement of tumors in this group in immune escape and suppression. In addition, TMB was also higher in the high-risk group. Although PD-L1 and TMB are considered valuable biomarkers for selecting patients for immunotherapy, their predictive performance has not been as expected. So far, there is consensus on detection methods and thresholds of PD-L1 and TMB [62]. Therefore, it is unclear whether PD-L1 expression levels and calculated TMB values in the TCGA database are suitable for predicting immunotherapy response.

The drug sensitivity analysis revealed that certain commonly used chemotherapeutic agents for lung cancer, including cisplatin, docetaxel, paclitaxel, and vinorelbine, exhibited lower IC50 values in the high-risk group. Conversely, some targeted inhibitors demonstrated better efficacy in the low-risk group. These findings imply that ECM plays an important role in influencing drug responses to chemotherapy, targeted therapy, and immunotherapy.

There are many prognosis signatures derived from high-throughput sequencing data, however, translating a bioinformatics signature into a clinically utilization is a complex process. In contrast to high-throughput sequencing used in various studies, the more commonly used methods for gene or protein expression level in clinical is qPCR and immunohistochemistry. However, this is influenced by the detection instruments, operators, and sample handling, making it difficult to establish a standardized performance benchmarks for prognostic tests. Therefore, further research is needed to develop new detection methods for convenient clinical utilization, such as liquid biopsy. It is essential to continue research and validation efforts to define acceptable sensitivity and specificity levels, specify the expected positive and negative predictive values and clarify how they impact clinical decision making. Moreover, a substantial number of clinical samples are still required to undertake a rigorous, well-controlled external validation that includes diverse patient cohorts.

Even though our risk score and nomogram can predict LUAD prognosis with good accuracy, their clinical utility is limited by gene expression assays. As a retrospective study, the data might not accurately represent the broader patient population for selection bias. Since most of the patients in the datasets had stage I-III LUAD, the performance of the model in advanced stage still needs to be validated. Missing or inaccurate data also limited the accuracy of the signature, and changes in treatment approaches limited the relevance of the signature to current clinical practice. Moreover, different datasets may have inherent variations in patient demographics, disease subtypes, and clinical treatments. Thus, this signature may not generalize well to other populations or clinical settings. Hence, prospective clinical studies are essential to validate the predictive efficacy of our risk assessment model. While we uncovered the association between the ECM risk score and the tumor immune microenvironment, the mechanism by which these ECM-related genes influence immune cells remains unclear and warrants further in vitro and in vivo studies. In conclusion, we developed a 4-gene signature derived from ECM-related genes and constructed and validated a nomogram for predicting the survival of LUAD patients across diverse datasets. Moreover, this 4-gene signature was associated with tumor immune microenvironment, infiltrated immune cell types, somatic mutations, and treatment response in LUAD patients. Stratifying patients using this signature could potentially aid clinicians in selecting tailored treatments for individual patients.

## Ethics approval

The study was conducted in accordance with the Declaration of Helsinki (as revised in 2013).The study was approved by the Human Research Ethics Committee of the Third Xiangya Hospital of Central South University(No. 23413, Jun 21, 2023).

## Funding

This work was supported by National Key R&D Program of China (No.2021YFC2500500), 10.13039/501100001809National Natural Science Foundation of China (grant number 82270519,81970252,81870352), Hunan Province Science and Health Union Foundation (grant number 2018JJ6135) and the Key Research and Development Project of Hunan Province (grant number 2020SK2087).

## Data availability statement

The datasets analyzed during the current study are available from the Cancer Genome Atlas (TCGA-LUAD, https://gdc.cancer.gov/) and the GEO database (https://www.ncbi.nlm.nih.gov/geo/, GSE116959, GSE19188, GSE19804, GSE30219, GSE32863, GSE40791, GSE43458, GSE75037, GSE72094, and GSE68465).All data generated during this study are available from the corresponding author on reasonable request.

## CRediT authorship contribution statement

**Yanfei Chai:** Writing – review & editing, Writing – original draft, Visualization, Validation, Methodology, Formal analysis, Data curation, Conceptualization. **Yuchao Ma:** Writing – review & editing, Visualization, Validation, Methodology, Formal analysis, Data curation. **Wei Feng:** Writing – review & editing, Visualization, Validation, Methodology, Formal analysis, Data curation. **Hong Xiang:** Writing – review & editing, Visualization, Validation, Methodology, Formal analysis, Data curation. **Hongwei Lu:** Writing – review & editing, Writing – original draft, Supervision, Software, Resources, Project administration, Investigation, Funding acquisition, Conceptualization. **Longyu Jin:** Writing – review & editing, Writing – original draft, Supervision, Software, Resources, Project administration, Investigation, Funding acquisition, Conceptualization.

## Declaration of competing interest

The authors declare that they have no known competing financial interests or personal relationships that could have appeared to influence the work reported in this paper.

## List of abbreviations

ECM: Extracellular Matrix
LUAD: Lung Adenocarcinoma
TCGA: The Cancer Genome Atlas
GEO: Gene Expression Omnibus
LASSO: Least Absolute Shrinkage and Selection Operator
NSCLC: Non-Small Cell Lung Cancer
OS: Overall Survival
TNM: Tumor-Node-Metastasis
TME: Tumor Microenvironment
TPM: Transcripts Per Million
DEG: Differentially Expressed Gene
FC: Fold Change
RRA: Robust Rank Aggregation
GO: Gene Ontology
KEGG: Kyoto Encyclopedia of Genes and Genomes
PPI: Protein–Protein Interaction
PFS: Progression-Free Survival
RCS: Restricted Cubic Splines
AIC: Akaike Information Criterion
PCA: Principal Component Analysis
t-SNE: t-Distributed Stochastic Neighbor Embedding
IDI: Integrated Discrimination Improvement
DCA: Decision Curve Analyses
AUC: Area Under the Curve
ROC: Receiver Operating Characteristics
GSEA: Gene Set Enrichment Analysis
TMB: Tumor Mutational Burden
TIDE: Tumor Immune Dysfunction and Exclusion
IPS: Immunophenoscore
IC50: the Half Maximal Inhibitory Concentration
qRT-PCR: Quantitative Real-Time PCR
