# Supplementary material for: Identification and validation of a 4-extracellular matrix gene signature associated with prognosis and immune infiltration in lung adenocarcinoma
Source: Heliyon. 2024 Jan 9;10(2):e24162. doi: 10.1016/j.heliyon.2024.e24162 (PMC10827462; doi:10.1016/j.heliyon.2024.e24162)
Supplement: Multimedia component 1 [file mmc1.pdf]

Supplementary Figures

Identification and validation of a 4-extracellular matrix gene signature associated with prognosis and immune infiltration in lung adenocarcinoma

Yanfei Chai [1,2], Yuchao Ma [2], Wei Feng [2], Hong Xiang [1,3], Hongwei Lu[1,3]*, Longyu Jin [2]*

***Corresponding Authors:**
Longyu Jin, jinlongyu1123@163.com; Hongwei Lu, hongweilu@csu.edu.cn

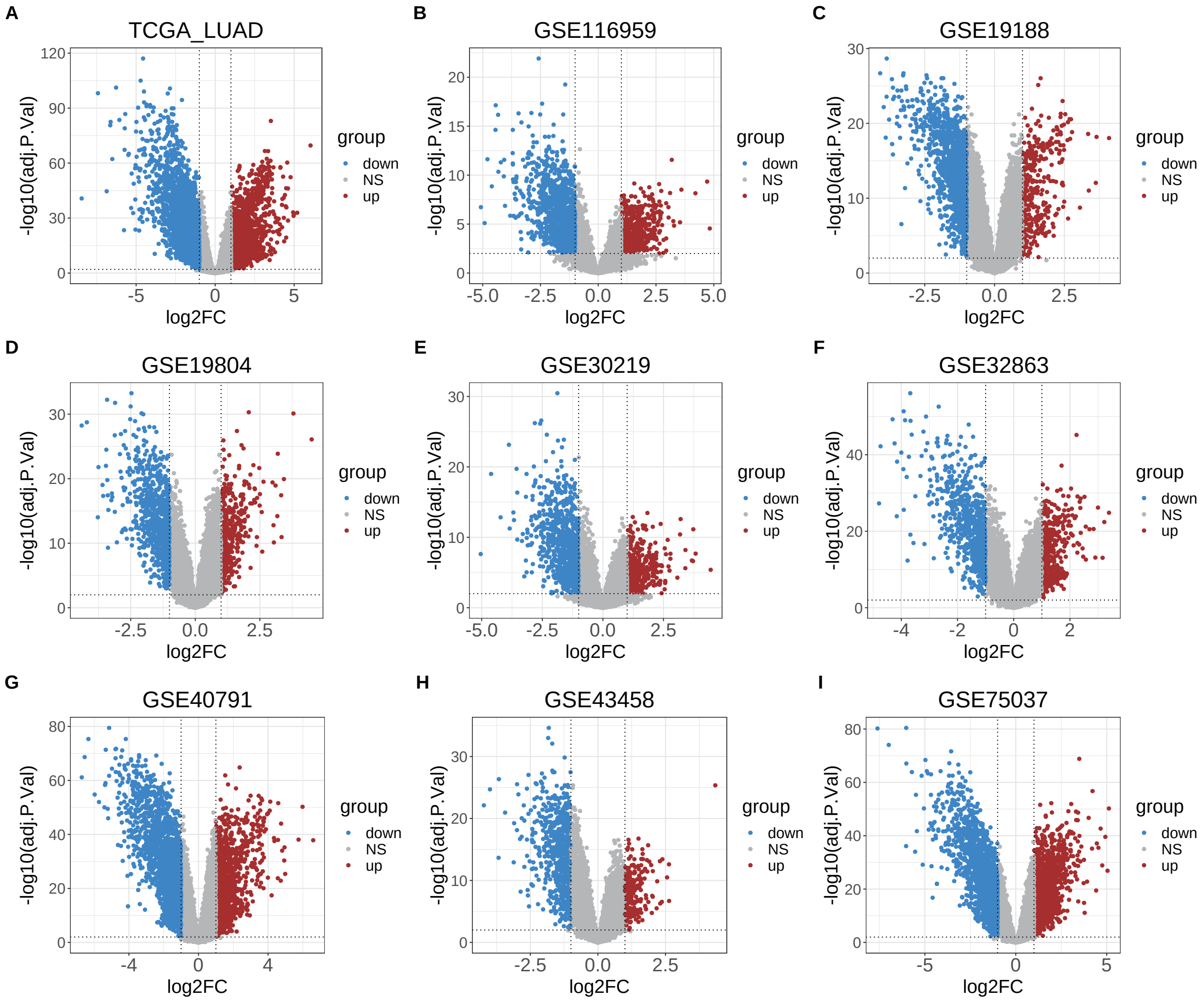

Figure S1.Volcano plot of the DEGs in nine datasets, including TCGA LUAD(A), GSE116959(B), GSE19188(C), GSE19804(D), GSE30219(E), GSE32863(F), GSE40791(G), GSE43458(H) and GSE75037(I).

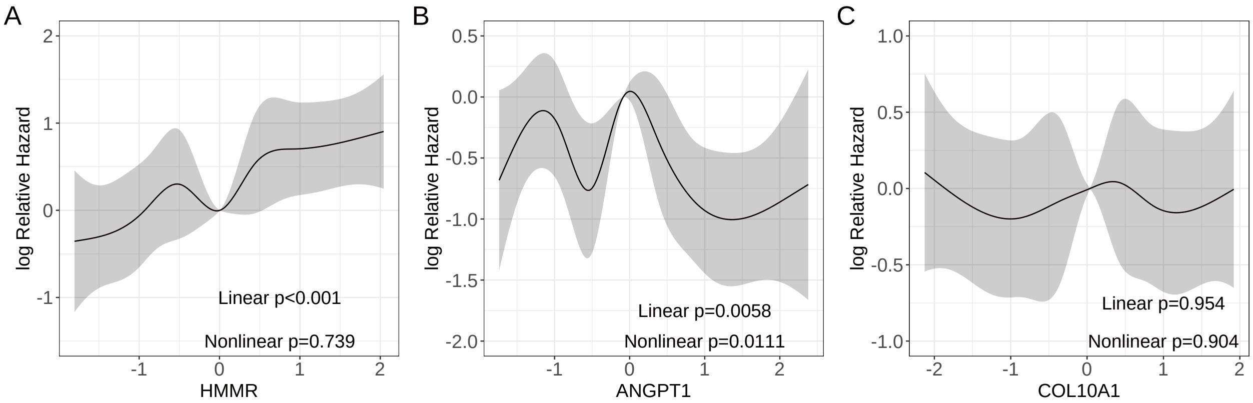

Figure S2. RCS curve indicated the linear effect of different genes on OS. HMMR**(A)** had a purely linear effect on OS, ANGPT1**(B)** had both linear and nonlinear effect on OS, and COL10A1**(C)** did not have linear effect on OS.

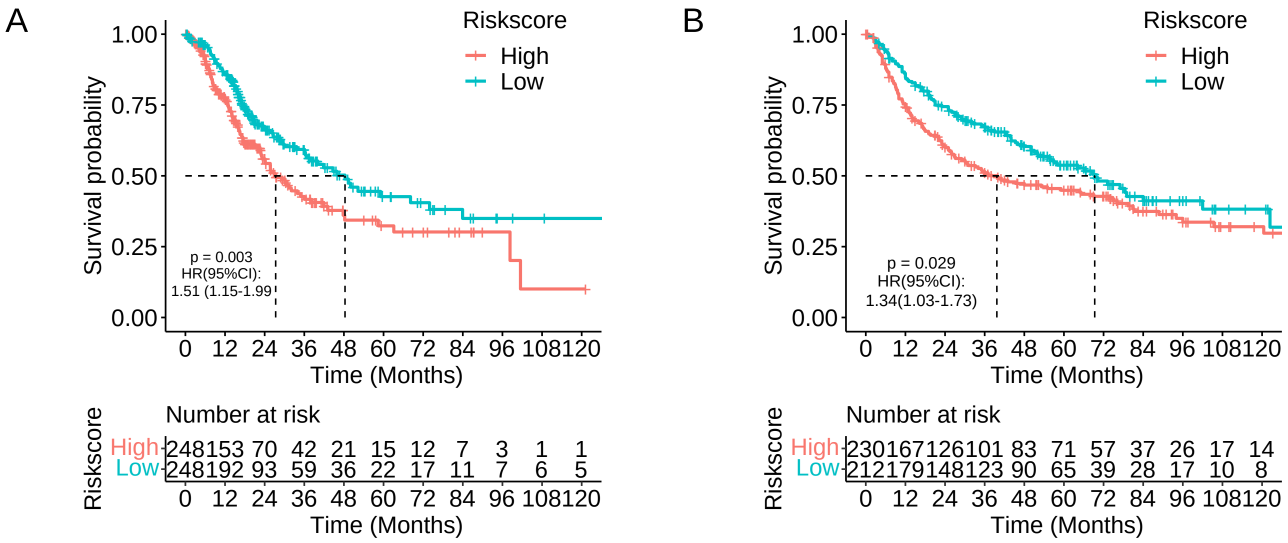

Figure S3. PFS was better in patients with low risk scores than those with high risk scores in TCGA dataset**(A)** and GSE68465**(B)**.

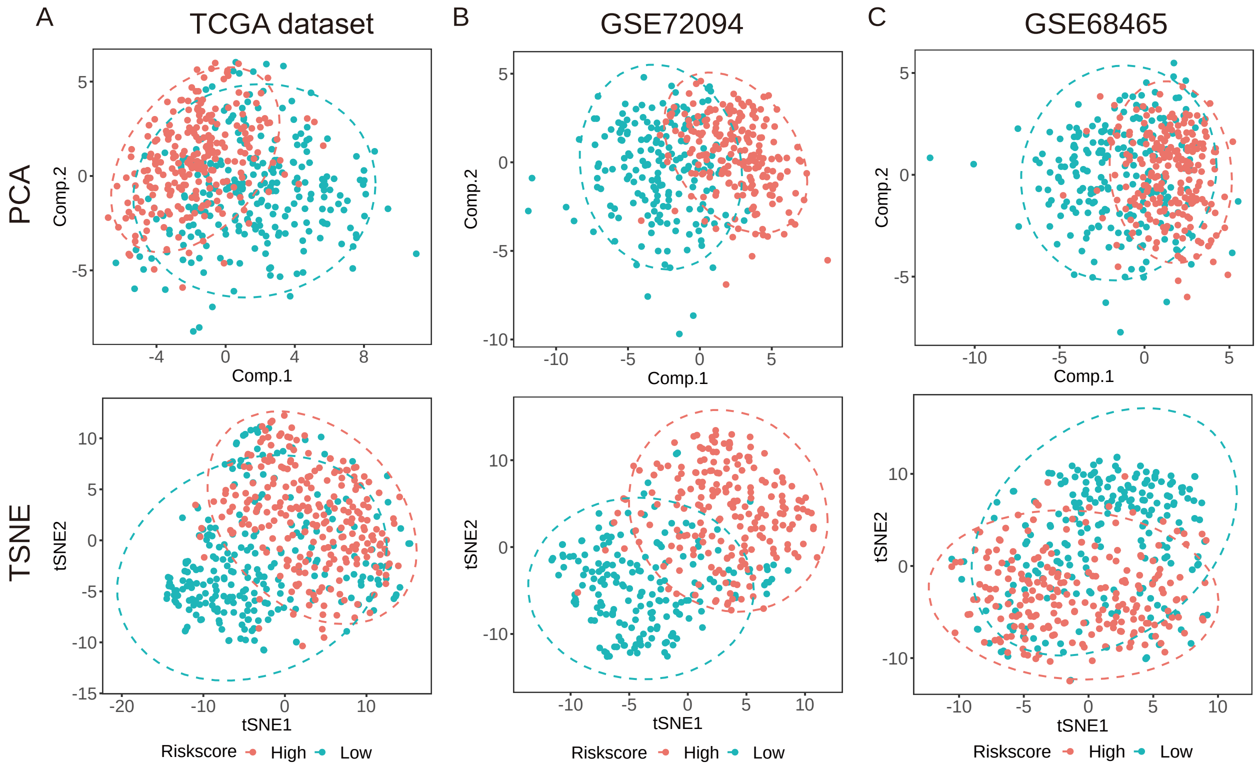

Figure S4. PCA and tSNE in TCGA dataset**(A)**, GSE72094**(B)** and GSE68465**(C).**

	Median OS			
	Low	High	HR(95%CI)	p
**All Patients**	**57.5**	**37.8**	**0.509 (0.376−0.689)**	**<0.001**
**Age**				
<65y	165.4	48.5	0.417 (0.274−0.633)	<0.001
>66y	55.1	34.2	0.576 (0.367−0.903)	0.016
**Sex**				
Female	53.3	37.8	0.565 (0.373−0.854)	0.007
Male	77.3	41.9	0.464 (0.295−0.730)	0.001
**Smoking**				
Yes	59.7	41.9	0.543 (0.392−0.752)	<0.001
No	49.7	34.2	0.330 (0.143−0.760)	0.009
**Lateral**				
Right	54.1	43.1	0.683 (0.465−1.003)	0.052
Left	59.9	29.3	0.320 (0.194−0.528)	<0.001
**T**				
T1	105.6	43.1	0.530 (0.290−0.969)	0.039
T2	54.1	42.9	0.560 (0.374−0.838)	0.005
T3−4	32.5	15.6	0.442 (0.210−0.930)	0.032
**N**				
N0	105.6	48.5	0.552 (0.360−0.845)	0.006
N1	38.2	30.7	0.682 (0.381−1.218)	0.196
N2−3	42.2	26.9	0.701 (0.347−1.418)	0.323
**M**				
M0	59.7	41	0.501 (0.366−0.686)	<0.001
M1	32.5	34.8	0.816 (0.243−2.734)	0.741
**Stage**				
I	105.6	87.3	0.624 (0.383−1.016)	0.058
II	41.2	34.9	0.643 (0.367−1.125)	0.122
III−IV	32.5	26.9	0.599 (0.336−1.069)	0.083

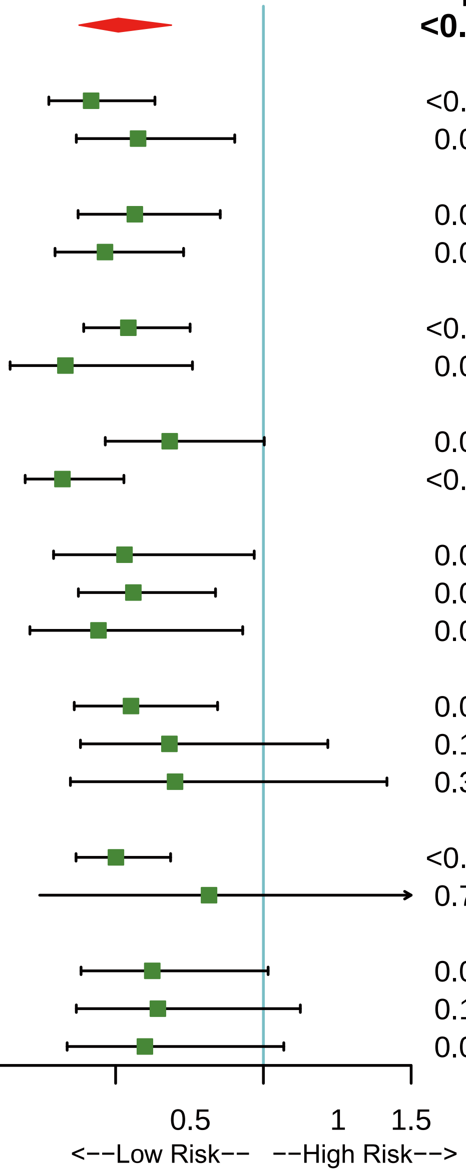

Figure S5. Subgroup analysis of overall survival between high and low risk group in TCGA dataset.

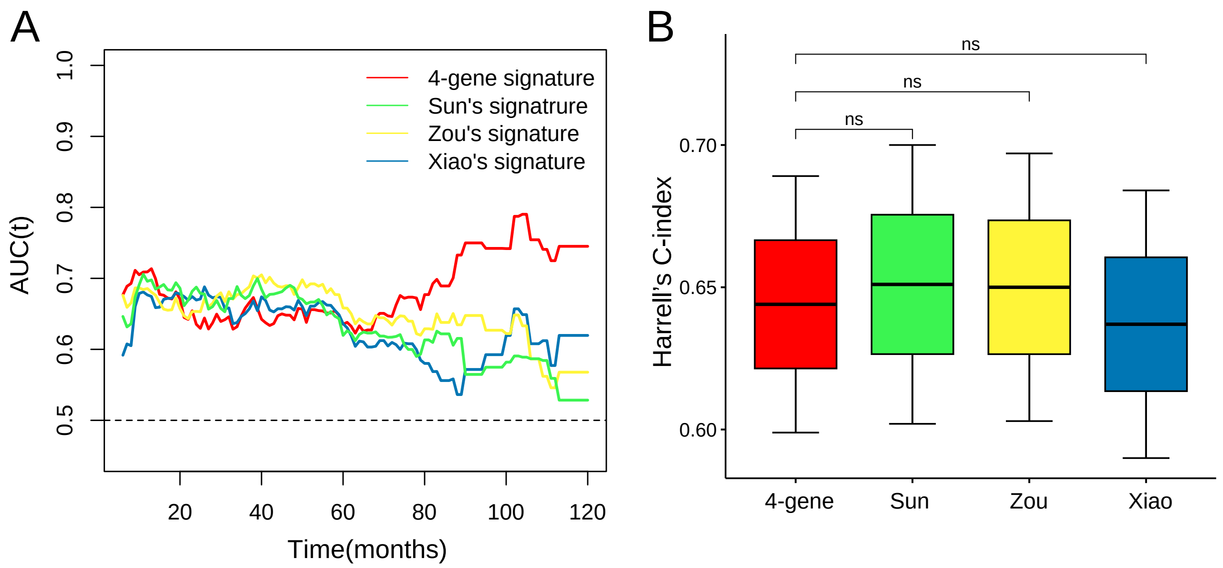

Figure S6. Comparison between 4-ECM signature and three published models. (A) time-ROC,(B) Harrell's C-index.

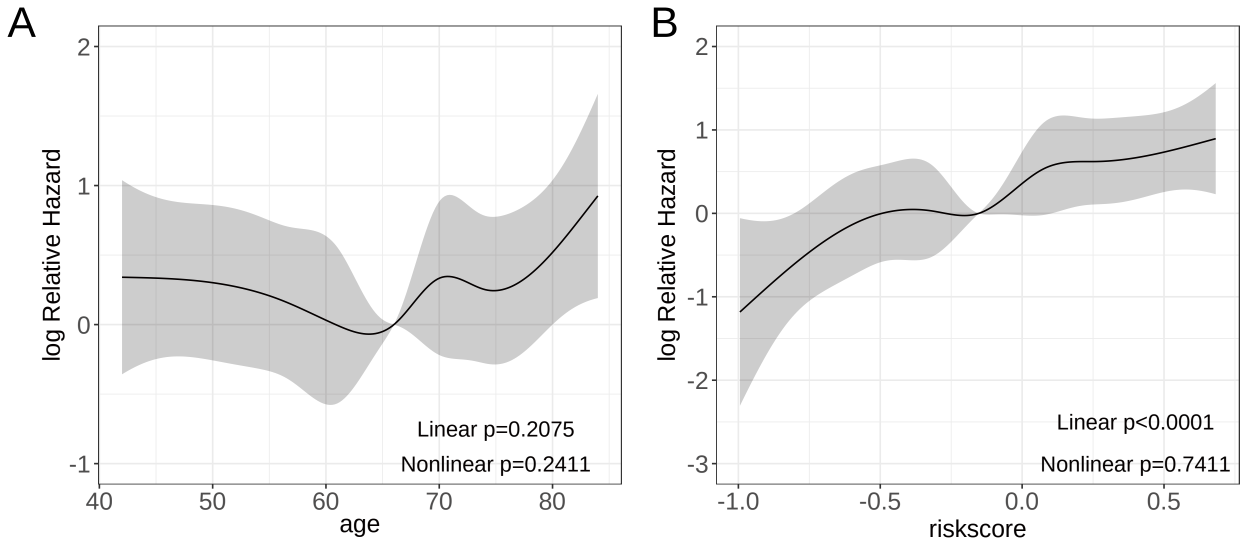

Figure S7. Linear effect of risk score**(A)** and age**(B)** on OS in RCS curve.

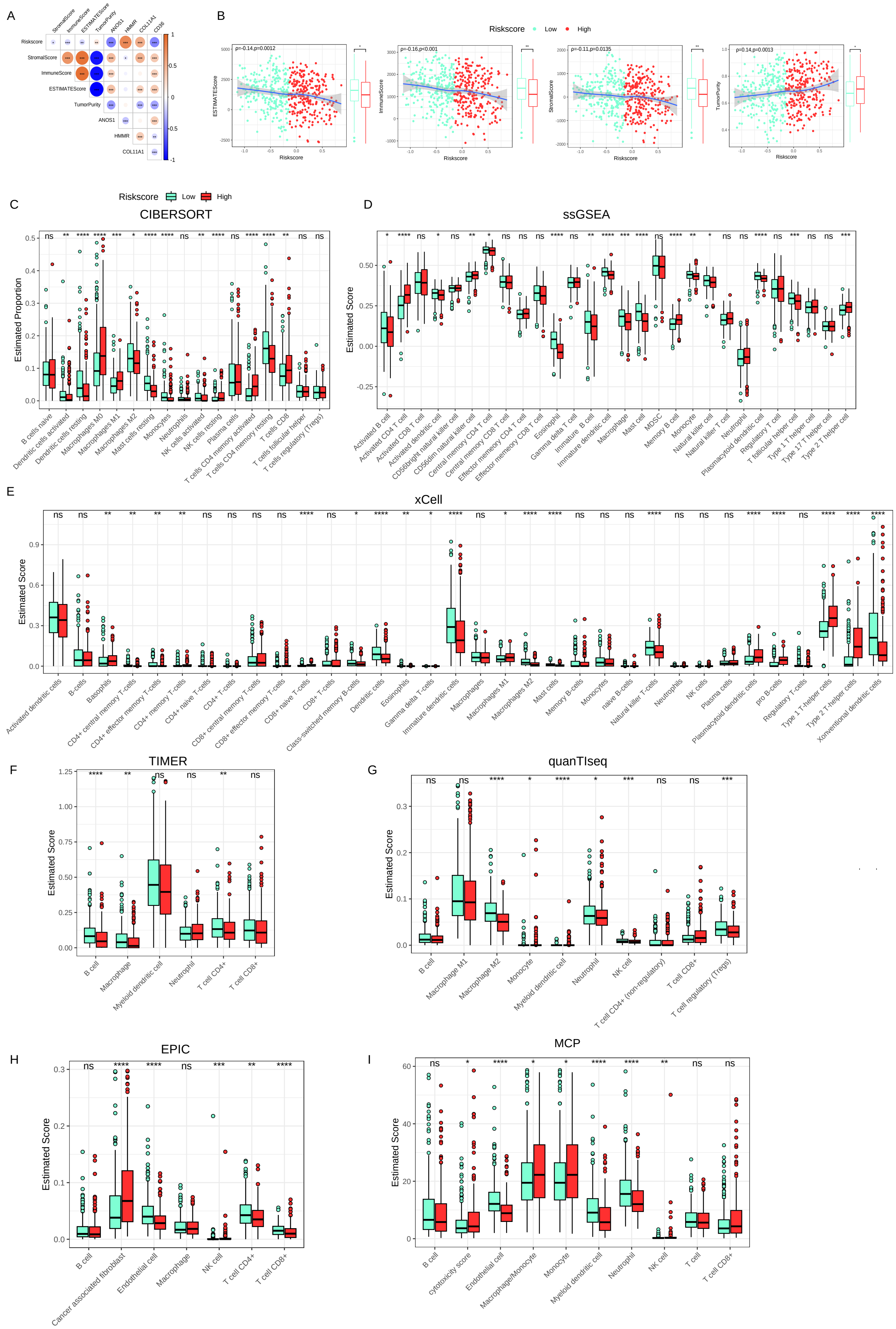

Figure S8. Correlation between risk score and tumor microenvironment.
(A) Correlation between risks core and ESTIMATE score, immune score, stromal score and tumor purity.
(B) Distribution of ESTIMATE score, immune score, stromal score and tumor purity between high and low risk group.
(C-I) Comparison of infiltrating immune cells in CIBERSORT(C), ssGSEA (D) , xCell (E), TIMER(F), quanTIseq(G), EPIC(H), MCP-counter(I) between high and low risk group.

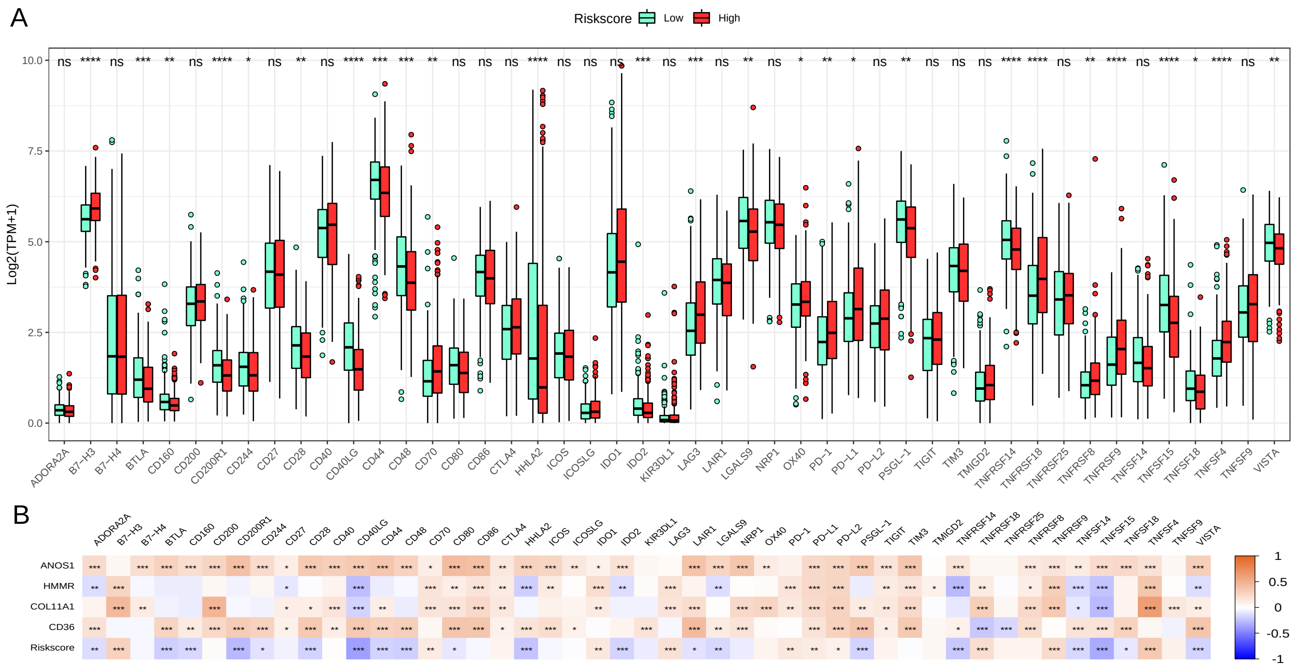

Figure S9. (A) Analyses for the expression of immune checkpoints between low and high risk group. **(B)** Correlation analysis for risk score and expression of immune checkpoints. * p < 0.05; ** p<0.01,*** p < 0.001,**** p<0.0001.

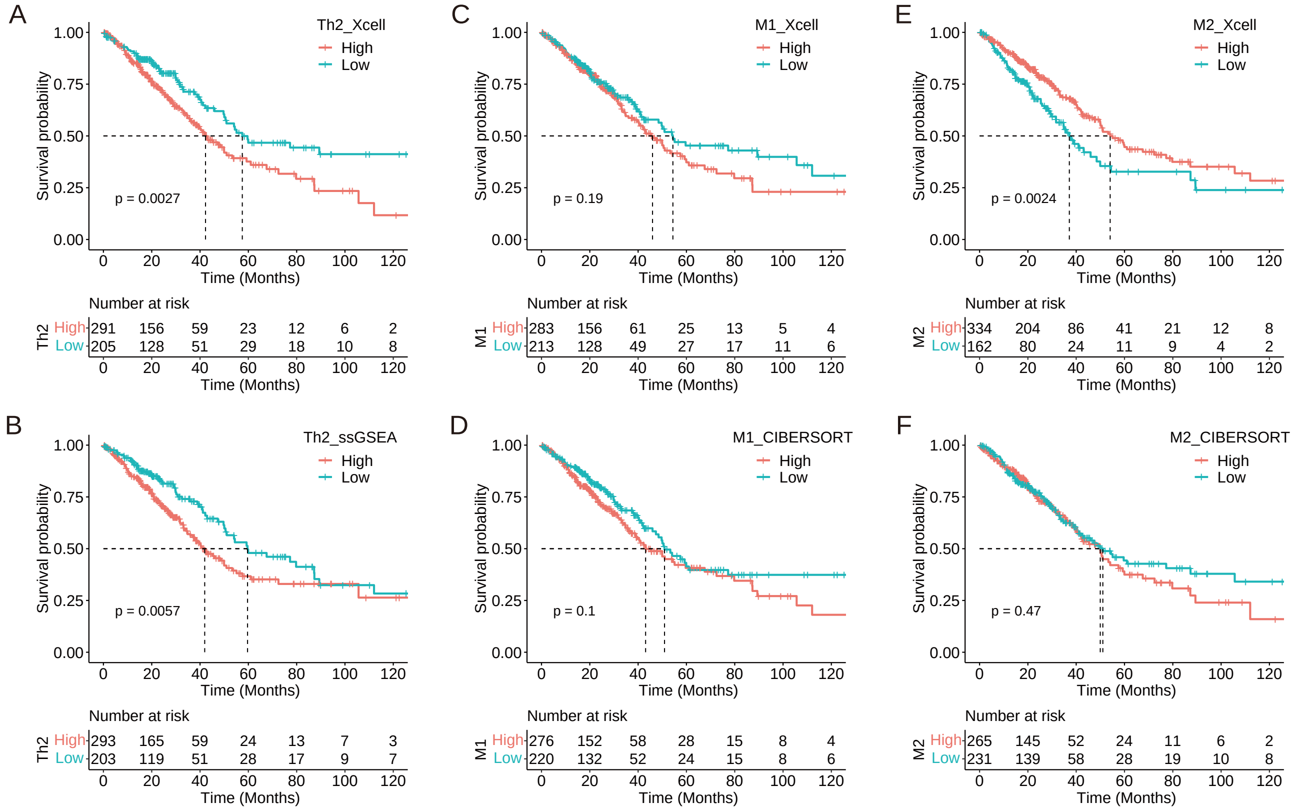

Figure S10. Kaplan-Meier survival analysis in TCGA cohort with different immune cell infiltration. **(A)** Th2 in xCell, **(B)** Th2 in ssGSEA, **(C)** M1 in xCell, **(D)** M1 in CIBERSORT, **(E)** M2 in xCell, **(F)** M2 in CIBERSORT.
